# Agronomic evaluation of shade tolerance of 16 spring *Camelina sativa* (L.) Crantz genotypes under different artificial shade levels using a modified membership function

**DOI:** 10.3389/fpls.2022.978932

**Published:** 2022-08-29

**Authors:** Yawen Wang, Jialin Yu, Yang Gao, Zhiwei Li, Do-Soon Kim, Min Chen, Yi Fan, Haixi Zhang, Xuebing Yan, Chuan-Jie Zhang

**Affiliations:** ^1^College of Animal Science and Technology, Yangzhou University, Yangzhou, Jiangsu Province, China; ^2^Peking University Institute of Advanced Agricultural Science, Weifang, Shandong, China; ^3^Department of Agriculture, Forestry, and Bioresources, Research Institute of Agriculture and Life Sciences, College of Agriculture and Life Sciences, Seoul National University, Seoul, South Korea; ^4^Henan Napu Biotechnology Co., Ltd., Zhengzhou, Henan Province, China; ^5^Research Center for Camelina sativa Planting and Engineering Technology, Anyang, Henan Province, China

**Keywords:** a modified membership function, camelina, intercropping, oil quality, shade response, shade tolerance

## Abstract

Camelina [*Camelina sativa* (L.) Crantz] is currently gaining considerable attention as a potential oilseed feedstock for biofuel, oil and feed source, and bioproducts. Studies have shown the potential of using camelina in an intercropping system. However, there are no camelina genotypes evaluated or bred for shade tolerance. The objective of this study was to evaluate and determine the shade tolerance of sixteen spring camelina genotypes (growth stage: BBCH 103; the plants with 4–5 leaves) for intercropping systems. In this study, we simulated three different shade levels, including low (LST), medium (MST), and high shade treatments (HST; 15, 25, and 50% reduction of natural light intensity, respectively), and evaluated the photosynthetic and physiological parameters, seed production, and seed quality. The mean chlorophyll pigments, including the total chlorophyll and chlorophyll *a* and *b* across the 16 genotypes increased as shade level increased, while the chlorophyll fluorescence parameter F_v_/F_m_, chlorophyll *a*/*b*, leaf area, the number of silicles and branches plant^−1^ decreased as shade level increased. The first day of anthesis and days of flowering duration of camelina treated with shade were significantly delayed and shortened, respectively, as shade increased. The shortened lifecycle and altered flowering phenology decreased camelina seed yield. Additionally, the shade under MST and HST reduced the seed oil content and unsaturated fatty acids, but not saturated fatty acids. The dendrograms constructed using the comprehensive tolerance membership values revealed that CamK9, CamC4, and ‘SO-40’ were the relatively shade-tolerant genotypes among the 16 camelina genotypes. These camelina genotypes can grow under the shade level up to a 25% reduction in natural light intensity producing a similar seed yield and seed oil quality, indicating the potential to intercrop with maize or other small grain crops. The present study provided the baseline information on the response of camelina genotypes to different shade levels, which would help in selecting or breeding shade-tolerant genotypes.

## Introduction

Camelina [*Camelina sativa* (L.) Crantz] is an industrial oilseed crop in the Brassicaceae family (Berti et al., 2016). Although camelina is not a well-known or widely cultivated crop, it has an ancient history dating back to 4,000 BCE (Knörzer, 1978; Berti et al., 2016). In the last decade, many studies in camelina genetics (Betancor et al., 2015; Bansal and Durrett, 2016), applications (Mafakher et al., 2022; Sarkhosh et al., 2022), agronomic management (Gesch, 2014; Berti et al., 2017; Zanetti et al., 2017; Walia et al., 2021), and ecological safety (Zhang and Auer, 2019, 2020; Zhang et al., 2020) have demonstrated the great interest and potential of this crop due to its multiple uses, such as feedstock for biofuel, food oil source, animal feed, and many more.

In China, camelina is a relatively new oilseed crop with an estimated planting area of about 500 ha in northern China (i.e., Gansu province, Xinjiang; Zhang et al., 2021). Recent agronomic evaluations showed that in milder climates in northern and eastern China, fall-seeded spring camelina genotypes well adapted in different environments with satisfactory seed and oil yields (Zhang et al., 2021; Gao et al., 2022a), shedding light on the great potential as an alternative oil source and for large-scale of this crop. It is worth mentioning that the camelina genotypes suitable for fall seeding are not necessarily the winter camelina type (Kuzmanović et al., 2021; Gao et al., 2022a). Winter camelina demonstrated completely different morphologies from spring camelina and has a strict requirement of vernalization to flower (Wittenberg et al., 2019; Zhang and Auer, 2019; Soorni et al., 2021, 2022).

One new potential use of camelina is using it in an intercropping system, such as maize (*Zea mays* L.)-soybean [*Glycine max* (L.) Merr.] (Berti et al., 2017). The advantage of the intercropping system includes increasing crop yield (Zhang et al., 2007), improving farmland use efficiency (Dhima et al., 2007), and reducing disease and pest damage (Sarrantonio and Gallandt, 2003; Banik et al., 2006). However, in this system, the shorter crops (i.e., soybean) suffered shade that resulted from the taller crops (i.e., maize) and received low amounts of sunlight for photosynthesis (Fan et al., 2018; Wen et al., 2020), which potentially affected the growth and development of the shorter crop (Wang et al., 2007, 2013). As a previous study reported, while intercropping of maize and soybean increased the economic benefits than that of sole soybean cropping (Yang et al., 2015), the seed quality and yield potential of soybean were significantly decreased due to the shade resulting from the maize (Wang et al., 2007, 2013).

To the best of our knowledge, while in an intercropping system intersowing camelina into pea (*Pisum sativum* L.), lupin (*Lupinus angustifolius* L.), or barley (*Hordeum vulgare* L.; Saucke and Ackermann, 2006; Paulsen, 2007; Leclère et al., 2019) were conducted previously in Germany and into the maize-soybean in the U.S. Midwest (North Dakota; Berti et al., 2017), all those camelina genotypes tested in the intercropping system were not evaluated for shade tolerance or originally selected or bred as an intercropped crop species. Therefore, the objective of this study was to evaluate and provide the baseline information on the shade tolerance of camelina genotypes. Firstly, the study was to evaluate (i) the shade tolerance of the 16 spring camelina genotypes under different artificial shade treatments; (ii) then, shade tolerance of those camelina genotypes were compared and determined using a modified membership function with 25 parameters measured (photosynthetic and physiological parameters, seed production, and seed oil quality); and (iii) the relative shade tolerance endured camelina genotypes were identified and discussed regarding its potential use in the intercropping system.

## Materials and methods

### Experimental site and seed source

A two-year experiment was conducted at the Yangzhou University Pratacultural Science Experimental Station, Yangzhou, Jiangsu Province (32°20’N, 119°23′E, 10 m a.s.l.) during 2020–2022. The experimental site is characterized by a humid subtropical climate and has a monsoon season from mid-June to the end of July. The meteorological data for the past 30-year (1981–2010) showed the yearly mean temperature and accumulated precipitation (1981–2010) are 15.7°C (highest: 20.2°C; lowest: 12.0°C) and 1,043 mm, respectively.^1^

In this study, 16 spring camelina genotypes were used with the basic information described in Table 1. Among them, 13 camelina genotypes (CamK1–CamK11, the origin of Korea; CamC2 and CamC3, the origin of China) were obtained from Yanbian University, Jilin province, China. Two camelina genotypes (CamC1 and CamC4, the origin of China) were obtained from the Research Center for *Camelina sativa* Planting and Engineering Technology at Anyang, Henan province, China. Additionally, one commercial camelina cultivar ‘SO-40’ obtained from Sustainable Oils, California, the U.S., was compared with the aforementioned 15 camelina genotypes. Laboratory seed germination tests for the 16 camelina genotypes showed greater than 95% seed germination for all genotypes.

**Table 1:** The origin and description of the 16 *Camelina sativa* used in this study.

Accession information	Country of origin	Seed source
CamK1	Korea	Yanbian University
CamK2	Korea	Yanbian University
CamK3	Korea	Yanbian University
CamK4	Korea	Yanbian University
CamK5	Korea	Yanbian University
CamK6	Korea	Yanbian University
CamK7	Korea	Yanbian University
CamK8	Korea	Yanbian University
CamK9	Korea	Yanbian University
CamK10	Korea	Yanbian University
CamK11	Korea	Yanbian University
CamC1	Jilin, China	RCCET^a^
CamC2	Jilin, China	Yanbian University
CamC3	Jilin, China	Yanbian University
CamC4	Jilin, China	RCCET
‘SO-40’	USA	Sustainable Oils, California

a RCCET: Research Center for *Camelina sativa* Planting and Engineering Technology.

### Experimental design and shade treatments

The 16 spring camelina genotypes were sown in 105-well plastic seedling trays (hole size: 3.5 × 1.5 × 4 cm) with organic horticultural potting soil (Yiyuan Agriculture and Forestry Ltd., China) in September 2020 and 2021 and grown in a greenhouse at Yangzhou University Pratacultural Science Experimental Station at 25°C with a 12/12 h photoperiod supplemented by an overhead sodium lamp. Young seedlings with 2–3 leaves were transplanted into plastic pots (height: 13.5 cm; diameter: 16.5 cm) containing the previously mentioned potting soil. Each plastic pot contained three well-grown seedlings of each camelina genotype. In mid-October 2020 and 2021, the 16 camelina genotypes with 4–5 leaves (BBCH 103: Single true leaf on the third node developed; Martinelli and Galasso, 2011) were moved outdoor for shade treatment (*n* = 3 pots for each genotype). Three different shade treatments were achieved using the shade structures built from the PVC (cube size: 1.8 × 1.5 × 1.5 m) covering the three different levels of light transmission polyethylene netting (Jurong Huanan Plastic Products Co. Ltd., China). The HOBO Pendant data logger determined approximately 15, 25, and 50% reduction in natural light intensity inside each shade structure (namely low shade treatment-LST, medium shade treatment-MST, and high shade treatment-HST, respectively). Non-shade treated (NST) camelina plants served as the control group. The irrigation was conducted when necessary to maintain adequate soil moisture. During the experimental period, light intensity and mean air temperature inside each shade structure were recorded by the HOBO Pendant data logger every 5 min (HOBO UA-002, Onset Computer Corporation, Bourne, MA, United States). Additionally, to minimize the variations of microclimate within the shade structure and the shade effect from the surrounding environment, the location of the shade structures and pots were changed periodically. The experiment was designed as a randomized complete block design with three replications for each shade level. The shade structures for each LST, MST, and HST were successively removed just before the end of the anthesis of camelina genotypes under each treatment. The plants were then returned to the greenhouse and well-raised.

### Measurements

Data on photosynthetic (leaf chlorophyll fluorescence and chlorophyll content) and physiological (plant height, leaf area, number of branches, and flowering phenology) performance of each camelina genotype under the LST, MST, and HST were collected.

The leaf chlorophyll fluorescence parameter F_v_/F_m_ [F_v_/F_m_ = (F_m_-F_0_)/F_m_, where F_0_ = minimum fluorescence of dark-adapted leaves and F_m_ = maximum fluorescence] of each camelina genotype under LST, MST, and HST was determined by a chlorophyll fluorescence metre (FP110-LM/D, Czech Republic) using a detection time of 2 s and emitting light of 650 nm wavelength with an intensity of 3,500 μmol photons m^−2^ s^−1^. The measurements were conducted on the three dark-adapted (using leaf clips for 30 min) camelina leaves from each pot. A total of 27 points measurements, each replicated pot with nine measurements, were made on each camelina genotype under each treatment. The measurements were repeated on those same selected leaves every 2 weeks. For chlorophyll content measurement, leaves of each camelina genotype with a similar growth status were sampled at the beginning (BBCH 600: First flower open), peak (BBCH 605: 50% of flowers open), and end of flowering (BBCH 609: Fruit set visible) to determine the chlorophyll content, respectively. The extraction of leaf chlorophyll using the dimethyl sulfoxide (DMSO) method was described in Hiscox and Israelstam (1979). Following the process developed by Fan (2016), the absorbance of the extract solution at 645 and 663 nm was measured using a spectrophotometer, and the total chlorophyll, chlorophyll *a* and *b* contents were determined accordingly.

For physiological measurement, the topmost leaf (*n* = 3) was selected to determine the leaf area of each camelina genotype at the aforementioned growth stage (BBCH 600, 605, AND 609) under each treatment using a leaf area tester (YT-YMJ-P photometric leaf area meter, Shandong Yun Tang Intelligent Technology Co., Ltd.). At maturity (BBCH 809: Nearly all siliques are ripe, and the crop is ready to be harvested), the number of branches and silicles plant^−1^ and plant height for each of the 16 camelina genotypes were determined. Silicles obtained from each treatment were manually threshed, and the resulting seeds were dried in a forced air oven at 40°C for 48 h and weighed to determine the seed yield (g). The flowering phenology-related data were also collected on the first, peak, and end of anthesis for each of those camelina genotypes under each treatment.

### Seed quality analysis

#### Seed oil content

The oil content of the resulting 16 camelina genotypes seeds was determined following the minor modified Soxhlet extraction method at the Laboratory of Grass Germplasm Resources Research and Utilization, Yangzhou University, China (Li et al., 2018). Prior to oil extraction, the seeds of each camelina genotype were dried for 1.5 h at 130°C in a forced-air oven, cooled for 20 min in a desiccator, and ground into fine powder. After that, a 0.5 g powder sample was accurately weighed, wrapped with fat-free filter paper, and placed into the Soxhlet device. Two hundred ml of anhydrous ether was added to the Soxhlet device to exact the seed oil for 12 h, following a re-drying of defatted samples at 45°C for another 12 h. The oil content was determined by comparing the difference in sample mass before (m_0_) and after (m_1_) extraction using the following [Eq. (1)]:


[1] $$\mathrm{oil content}\;\left(\%\right)=\frac{m_{0}-m_{1}}{m_{0}}\times 100\%$$

#### Seed fatty acid profiles

The seeds of the 16 camelina genotypes from the 2020–2021 experiment were used to determine the seed fatty acid composition at the Animal Nutrition and Feed Engineering Technology Research Laboratory, Yangzhou University, China. Fatty acid methyl esters (FAMEs) were prepared following the method described by Augustin et al. (2019). The FAMEs profiles of camelina seed oil were analyzed by gas chromatography (GC; GC9800, Shanghai Kechuang Chromatographic Instrument Co., Ltd., China; flame ionization detector) equipped with an HP-88 capillary column (0.25 mm × 100 m × 0.2 μm, J and W, Folsom, CA, United States). The GC was set with an injection temperature of 270°C and detection temperature of 280°C. Helium as carrier gas was set at the flow rate of 1 ml min^−1^. The initial temperature of the GC was programed at 100°C for 13 min, increased by 10°C min^−1^ to 180°C for 6 min, 1°C min^−1^ to 200°C for 20 min, and final 4°C min^−1^ to 230°C for 35 min. The quantity of each FAME was determined by integrating the corresponding FAME peak and compared with a standard curve developed from a 37 FAMEs standard mixture (Nu-Chek Prep. Inc., Elysian, MN, United States). The content of individual fatty acids (%) was computed as the percentage of total fatty acid content.

### Statistical analysis and formula definition

Initially, prior to the analysis of variance (ANOVA), all data obtained from the current study were tested for normality using the Shapiro–Wilk test and showed normal distribution of residuals. The homogeneity of variances for the data was checked by Levene’s test. Then, the data were subjected to ANOVA using the statistical software R (version R i386 4.0.3) to determine the main effects (years, camelina genotypes, and shade levels) and interactions on photosynthetic (i.e., leaf chlorophyll fluorescence), physiological (i.e., plant height, leaf area), and seed oil-related (i.e., seed oil composition) parameters. As the ANOVA showed a significant treatment (i.e., year) effect, data for each year were analyzed separately, and mean values were presented for those parameters. When ANOVA revealed statistically different means, the Tukey *post-hoc* test was conducted to separate means (*p* ≤ 0.05).

To evaluate the level of shade tolerance of the 16 camelina genotypes tested, the shade tolerance coefficient [Eq. (2)], comprehensive shade tolerance coefficient [Eq. (3)], seed yield shade tolerance coefficient [Eq. (4)], and shade tolerance index [Eq. (5)] were calculated using all parameters measured following Blum and Jordan (1985), Liu et al. (2017), and Du et al. (2011) as below:


[2] $$STC_{pg}=V_{pgst}/V_{pgns}$$

[3] $$CSTC_{g}=\frac{1}{n}\sum_{t=1}^{n}STC_{pg}$$

[4] $$YSTC_{g}=SYPP/\bar{SYPP}\;$$

[5] $$STI_{g}=CSTC_{g}\times YSTC_{g}$$

where $STC_{pg}$ and $V_{pgst}\;$represent the shade tolerance coefficient and values of the parameter (*p*) for the 16 camelina genotypes (*g*) determined for the three different shade treatments, respectively. $V_{pgns}$ is the value of parameters for non-shaded plants (*ns*). $CSTC_{g}$ is the comprehensive shade tolerance coefficient of the sixteen camelina genotypes, and *n* is the total number of parameters measured. $YSTC_{g}$ is the seed yield shade tolerance coefficient. SYPP and $\bar{SYPP}$ represent the seed yield of each camelina genotype and the average of the 16 camelina genotypes (yield plant^−1^) under the aforementioned shade treatments. $STI_{g}$ is the shade tolerance index of the 16 camelina genotypes (*g*).

To further reveal the ranks of the shade tolerance levels for the 16 camelina genotypes, a modified equation developed by Zadeh (1965) and Liu et al. (2017) was used to calculate the values of the membership function for each treatment:


[6] $$M_{pg}=\frac{STC_{pg}-STC_{pmin}}{STC_{pmax}-STC_{pmin}}$$

[7] $$C_{g}=\sum_{t=1}^{n}\left[M_{pg}\times \left(\left|r_{p}\right|/\sum_{t=1}^{n}\left|r_{p}\right|\right)\right]$$

where $M_{pg}$ is the membership function value of each parameter measured (*p*) for each camelina genotype (*g*);$\;STC_{pmin}$ and $STC_{pmax}$ are the minimum and maximum values of shade tolerance coefficients of each parameter, respectively; $C_{g}$ is the comprehensive membership function value for each camelina genotype under each treatment; $r_{p}$ is the correlation coefficient between the shade tolerance coefficient of parameter (*p*) and shade tolerance index of each camelina genotype (*g*). Finally, the value of the comprehensive tolerance membership function for each camelina genotype ($CT_{g}$) was calculated taking into account the performance of each genotype under those three treatments using [Eq. (8)].


[8] $$CT_{g}=\frac{1}{m}\sum_{t=1}^{m}\left[\frac{C_{g}}{\left(\frac{1}{ng}{\sum^{\text{​}}}_{t=1}^{ng}C_{g}\right)}\right]$$

where *m* and *ng* represent the number of shade treatments (*m* = 3) and camelina genotypes tested in this study (*ng* = 16), respectively. Based on the values, cluster analysis was conducted to classify the shade tolerance of the 16 camelina genotypes using SPSS soft version 21.0 (Chicago, IL, United States). The shade tolerance values were calculated above in this section using Microsoft Excel 2019.

## Results

### Light intensity and air temperature in different shade treatments

During the experimental period between 2020 and 2022, the total light intensity and biweekly mean temperature in each shade treatment were recorded and described in Supplementary Table 1. Through the experimental period, the sum of the yearly total light intensity for NST was 18900.2 lum/ft^2^, and consistently decreased with increasing the shade levels (LST: 15946.8 lum/ft^2^; MST: 14035.7 lum/ft^2^; HST: 8559.1 lum/ft^2^). The rate of light reduction for the three treatments compared to NST was approximately 15.6% (LST), 25.7% (MST), and 54.7% (HST), which was almost equivalent to the designed rate of light reduction for those structures (Supplementary Table 1). Additionally, a similar temperature pattern within the shade structures was shown with the higher mean temperature observed for the LST than for those of MST and HST. As shown in Supplementary Table 1, the mean temperature across the entire recording period was 12.3°C for the LST, which was higher than that of the greater shade levels (11.9 and 11.2°C for MST and HST, respectively). The average cumulated precipitation during the experimental period was 135.4 mm, which was generally comparable to the value of 146.3 mm for the past 30-year average (Supplementary Table 1).

### Effect of shade on camelina photosynthetic and physiological parameters and seed production

**Figure 1:**
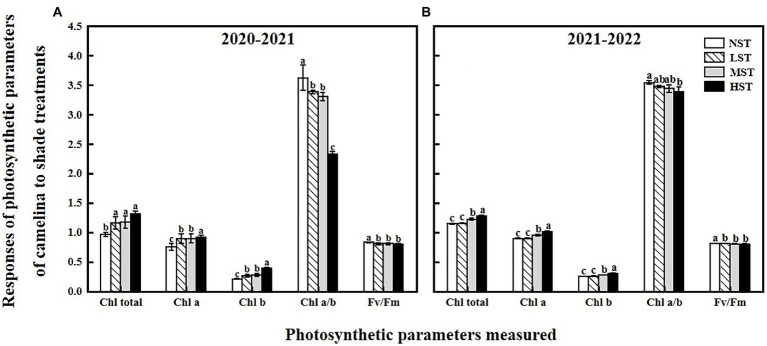
Responses of photosynthetic parameters, including the total chlorophyll content (mg g^−1^), chlorophyll *a* (mg g^−1^), chlorophyll *b* (mg g^−1^), chlorophyll *a*/*b*, and F_v_/F_m_ of camelina across the 16 genotypes to three different artificial shade levels (15%-LST, 25%-MST, and 50%-HST reduction in natural light intensity, respectively) in 2020–2021 **(A)** and 2021–2022 **(B)**. Individual values are means of each parameter measured across the 16 camelina genotypes ± stand errors. Different letters represent the significant different values by Tukey *post-hoc* test (*p* ≤ 0.05).

**Figure 2:**
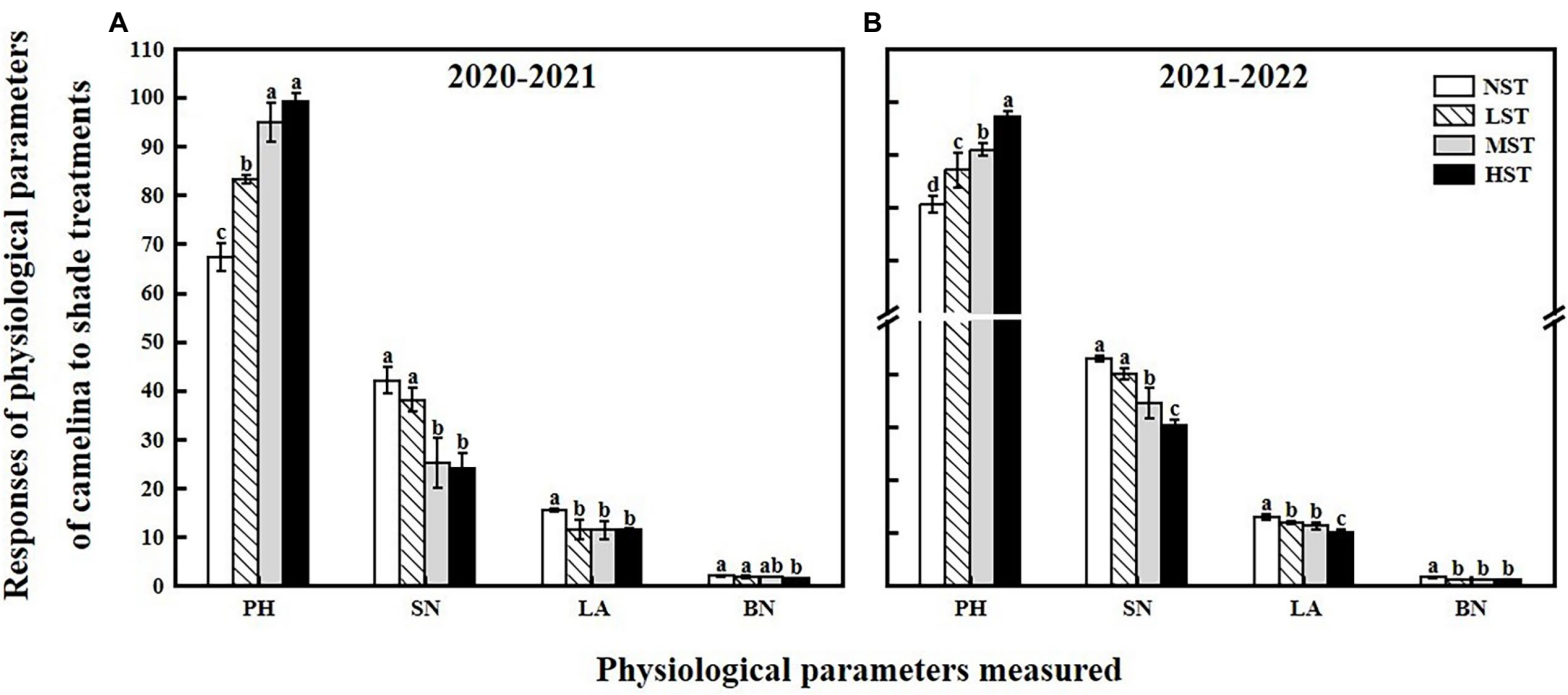
Responses of physiological parameters, including the plant height (PH, cm), the number of silicles plant^−1^ (SN), leaf area (LA, cm^2^), and the number of branches plant^−1^ (BN) of camelina across the 16 genotypes to three different artificial shade levels (15%-LST, 25%-MST, and 50%-HST reduction in natural light intensity, respectively) in 2020–2021 **(A)** and 2021–2022 **(B)**. Individual values are means of each parameter measured across the 16 camelina genotypes ± stand errors. Different letters represent the significant different values by Tukey *post-hoc* test (*p* ≤ 0.05).

As a significant treatment effect (i.e., year) was shown, data of those parameters measured were presented separately for each year. Overall, all the three factors (year, camelina genotype, and shade level) significantly (*p < 0.05*) affected the photosynthetic and physiological parameters, and seed production of camelina genotypes under each treatment (Table 2). A consistent trend of variation in those parameters measured was shown for both experimental years. For example, in both years, all the three shade treatments (LST, MST, and HST) significantly (*p < 0.05*) increased the content of total chlorophyll (range of mean: 0.96–1.31 and 1.14–1.28 mg g^−1^ for 2020–2021 and 2021–2022, respectively), chlorophyll *a* (range of mean: 0.75–0.92 and 0.89–1.00 mg g^−1^ for 2020–2021 and 2021–2022, respectively), chlorophyll *b* (range of mean: 0.21–0.39 and 0.25–0.30 mg g^−1^ for 2020–2021 and 2021–2022, respectively), and plant height (range of mean: 67.3–99.2 and 80.4–97.1 cm for 2020–2021 and 2021–2022, respectively) of camelina genotypes tested compared to NST (Figures 1, 2). By contrast, the shade treatments reduced the values of F_v_/F_m_ (range of mean: 0.80–0.83 and 0.80–0.81 for 2020–2021 and 2021–2022, respectively), chlorophyll *a*/*b* (range of mean: 2.33–3.62 and 3.38–3.54 for 2020–2021 and 2021–2022, respectively), leaf area (range of mean: 11.4–15.5 and 10.0–13.0 for 2020–2021 and 2021–2022, respectively), the number of silicles plant^−1^ (range of mean: 24.1–42.0 and 30.5–43.0 for 2020–2021 and 2021–2022, respectively), and the number of branches plant^−1^ (range of mean: 1.5–2.0 and 1.0–1.5 for 2020–2021 and 2021–2022, respectively) of camelina genotypes (Figures 1, 2).

**Table 2:** Effect of fixed sources of variation on photosynthetic and physiological parameters and seed production of camelina grown under different shade treatments.

Source													
Photosynthetic parameters	Chl T			Chl a		Chl b		Chl a/b		Fv/Fm			
	DF	MS	*p*	MS	*p*	MS	*p*	MS	*p*	MS	*p*	–	–
Year (Y)	1	0.226	^***^	0.488	^***^	0.022	^***^	8.691	^***^	0.002	^*^	–	–
Camelina genotype (CG)	15	0.100	^***^	0.050	^***^	0.004	^***^	0.361	^***^	0.001	^***^	–	–
Shade level (SL)	3	0.955	^***^	0.335	^***^	0.235	^***^	9.481	^***^	0.006	^***^	–	–
Y × CG	15	0.172	^***^	0.114	^***^	0.006	^***^	0.326	^***^	0.001	^***^	–	–
Y × SL	3	0.222	^***^	0.080	^***^	0.076	^***^	6.323	^***^	0.002	^**^	–	–
CG × SL	45	0.130	^***^	0.085	^***^	0.007	^***^	0.515	^***^	<0.001	NS^a^	–	–
Y × CG × SL	45	0.207	^***^	0.124	^***^	0.012	^***^	0.522	^***^	<0.001	NS	–	–
Residuals	256	0.011	–	0.007	–	0.001	–	0.098	–	<0.001	–	–	–
Physiological parameters and seed productions													
	PH			LA		BN		SN		SY		SOC	
	DF	MS	*p*	MS	*p*	MS	*p*	MS	*p*	MS	*p*	MS	*p*
Year (Y)	1	11,422	^***^	76.09	^**^	26.235	^***^	2,146	^***^	0.180	^**^	0.003	NS
Camelina genotype (CG)	15	1,556	^***^	60.28	^***^	0.811	^***^	2,203	^***^	0.223	^***^	0.011	^*^
Shade level (SL)	3	1,595	^***^	229.68	^***^	3.927	^***^	5,125	^***^	0.394	^***^	0.145	^***^
Y × CG	15	197	^**^	68.57	^***^	0.357	NS	627	^***^	0.067	^***^	0.013	^**^
Y × SL	3	1,089	^***^	38.57	^*^	0.152	NS	375	NS	0.042	NS	0.035	^***^
CG × SL	45	253	^***^	29.77	^***^	0.212	NS	313	^*^	0.026	NS	0.010	^**^
Y × CG × SL	45	252	^***^	22.72	^***^	0.212	NS	223	NS	0.024	NS	0.006	NS
Residuals	256	87	–	10.26	–	0.239	–	192	–	0.022	–	0.005	–

Chl T (total chlorophyll content, mg/g); Chl a (chlorophyl a, mg/g); Chl b (chlorophyl b, mg/g); Chl a/b (chlorophyll a/b ratio); Fv/Fm (optimal/maximal photochemical efficiency of PSII in the dark); PH (plant height, cm); LA (leaf area, cm^2^); BN (number of branches plant^−1^); SY (seed yield per plant^−1^, g); SN (number of silicles plant^−1^); SOC (seed oil content).

*, **, and *** represent significant at 0.05, 0.01, and 0.001 probability level, respectively.

a NS: Not significantly different.

Different shade levels significantly (*p < 0.05*) delayed the first day of anthesis of camelina genotypes tested in this study (Supplementary Figure 1). Compared to the date for the first day of anthesis of camelina genotypes under NST, the mean delayed day was 10.5 d (range of mean: 9–12 d) for LST and 11.8 d (range of mean: 9–15 d) for MST. By contrast, the mean delayed days for the first day of anthesis of camelina genotypes under HST was about 29 d. Additionally, shade treatment shortened the mean flowering duration of camelina genotypes tested. For example, during the year 2020–2021, the mean flowering duration across the 16 camelina genotypes for NST was 43 d (19 Nov. 2020–1 Jan. 2021), decreased to 41 d (30 Nov. 2020–12 Jan. 2021) and 39 d (4 Dec. 2020–12 Jan. 2021) for LST and MST, respectively. Under HST, the mean flowering duration for the camelina genotypes tested was shorter about 1 week (36 d: 28 Dec. 2020–2 Feb. 2021) than that of NST. A similar pattern of flowering duration for those genotypes was also observed for 2021–2022 (Supplementary Figure 1).

**Figure 3:**
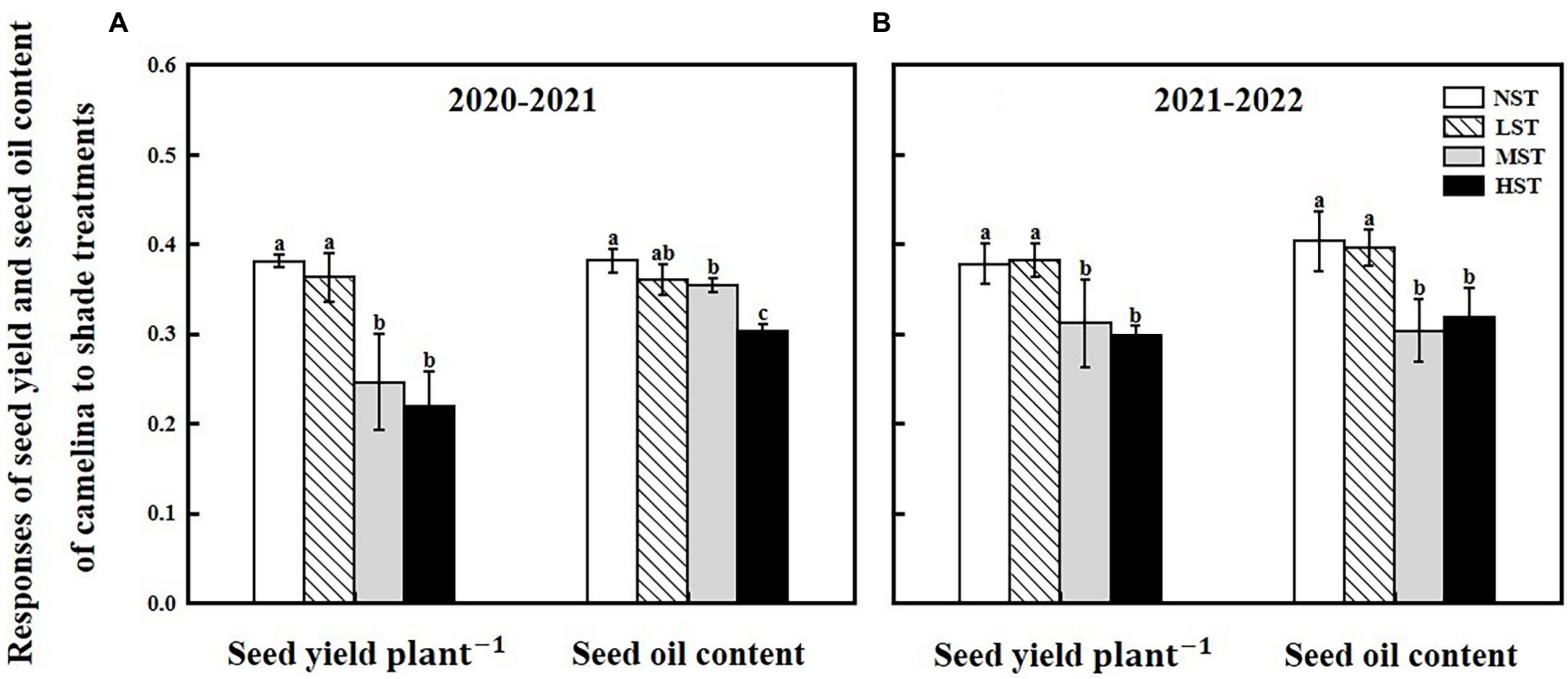
Responses of seed yield plant^−1^ (g) and seed oil content (%) of camelina across the 16 genotypes to three different artificial shade levels (15%-LST, 25%-MST, and 50%-HST reduction in natural light intensity, respectively) in 2020–2021 **(A)** and 2021–2022 **(B)**. Individual values are means of each parameter measured across the 16 camelina genotypes ± stand errors. Different letters represent the significant different values by Tukey *post-hoc* test (*p* ≤ 0.05).

Camelina seed yield was affected by year, camelina genotype, and shade level (Table 2). In 2020–2021, although the mean seed yield plant^−1^ across the 16 camelina genotypes for LST (mean: 0.36 g; range: 0.11–0.65 g) was statistically similar to that of NST (mean: 0.38 g; range: 0.14–0.70 g), it was significantly reduced by 35.5% (mean: 0.23 g; range: 0.07–0.50 g) and 42.7% (mean: 0.22 g; range: 0.05–0.49 g) for MST and HST, respectively (Figure 3). In 2021–2022, a similar trend in seed yield for those camelina genotypes under the three treatments was also observed in 2021–2022.

### Effect of shade on camelina seed oil quality

The ANOVA revealed that camelina genotype and shade level, but not year greatly (*p < 0.05*) affected the camelina seed oil content (Table 2). The mean seed oil content of camelina across year and the 16 camelina genotypes for LST (mean: 37.8%; range: 30.8–44.0%) was statistically equivalent to the NST (mean: 39.2%; range: 32.4–44.7%), and much greater than the values for MST (mean: 32.9%; range: 24.5–40.5%) and HST (mean: 31.0%; range: 23.5–37.8%; Figure 3).

**Figure 4:**
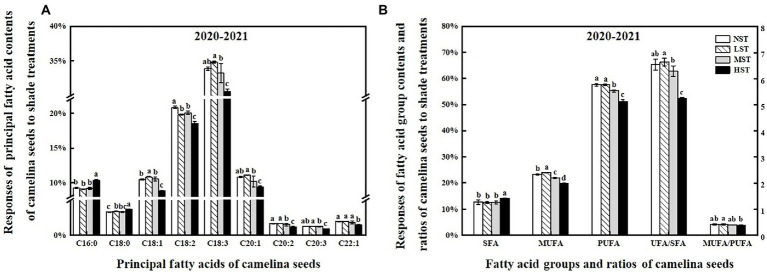
Responses of the principal fatty acid contents (%) **(A)**, the sum of the different fatty acid groups (%), the ratio of UFA/SFA and MUFA/PUFA **(B)** of camelina across the 16 genotypes to three different artificial shade levels (15%-LST, 25%-MST, and 50%-HST reduction in natural light intensity, respectively) in 2020–2021. Individual values are means of each parameter measured across the 16 camelina genotypes ± stand errors. Different letters represent the significant different values by Tukey *post-hoc* test (*p* ≤ 0.05). C16:0 (palmitic acid); C18:0 (stearic acid); C18:1 (oleic acid); C18:2 (linoleic acid); C18:3 (linolenic acid); C20:1 (eicosenoic acid); C20:2 (eicosadienoic acid); C20:3 (eicosatrienoic acid); C22:1 (erucic acid); SFA (saturated fatty acid); MUFA (monounsaturated fatty acid); PUFA (polyunsaturated fatty acid); UFA/SFA ratio = The sum of unsaturated fatty acid/the sum of saturated fatty acid; MUFA/PUFA ratio = The sum of mono-unsaturated/the sum of poly-unsaturated.

To reveal the shade effect on the principal fatty acid composition and content of camelina seed oil, the seed of each camelina genotype obtained from each treatment was analyzed for their fatty acid profiles. The ANOVA showed that genotype, shade level, and the interactions between them significantly (*p < 0.001*) affected the principal fatty acid contents (i.e., C16:0, C20:1), fatty acid groups (i.e., SFA, MUFA), and fatty acid ratios (ratios of unsaturated/saturated and mono-unsaturated/poly-unsaturated fatty acids; Table 3). While the shade treatment showed the different affecting patterns on individual fatty acid contents, overall, LST and MST hardly affected the saturated and unsaturated fatty acid contents compared to the NST (Figure 4). In contrast, HST significantly (*p < 0.05*) increased the saturated fatty acids contents (i.e., C16:0) but reduced the unsaturated fatty acids contents (i.e., C18:1, C18:2) compared to the NST, LST, and MST (Figure 4). For example, the contents of saturated fatty acid C16:0 and C18:0 for HST were 10.3 and 3.7%, respectively, which was significantly greater than the values of 9.1–9.3% and 3.3–3.4% for NST, LST, and MST (Figure 4A). However, unsaturated fatty acid contents, such as C18:2, C18:3 were significantly reduced to 18.5 and 30.5% for HST, respectively, compared to 19.8–20.8%, 33.2–34.8% for other treatments (Figure 4A). Additionally, HST significantly (*p < 0.05*) decreased the ratios of the sum of unsaturated/saturated and mono-unsaturated/poly-unsaturated fatty acids, whereas LST and MST almost did not change the ratios of those fatty acid groups (Figure 4B).

**Table 3:** Effect of fixed sources of variation on camelina seeds of principal fatty acid contents, fatty acid group contents and fatty acid ratios grown under different shade treatments.

Source											
Saturated fatty acid content	C16:0			C18:0		SFA		U/S ratio			
	DF	MS	*p*	MS	*p*	MS	*p*				
								MS	*p*		
Camelina genotype (CG)	15	0.001	^***^	<0.001	^***^	0.002	^***^	10.714	^***^	–	–
Shade level (SL)	3	0.002	^***^	<0.001	^***^	0.003	^***^	19.780	^***^	–	–
CG × SL	45	<0.001	^***^	<0.001	^***^	<0.001	^***^	2.256	^***^	–	–
Residuals	128	<0.001	–	<0.001	–	<0.001	–	0.000	–	–	–
Monounsaturated fatty acid content	C18:1			C20:1		C22:1		MUFA		MU/PU ratio	
	DF	MS	*p*	MS	*p*	MS	*p*	MS	*p*	MS	*p*
Camelina genotype (CG)	15	<0.001	^***^	0.002	^***^	<0.001	^***^	0.005	^***^	0.008	^***^
Shade level (SL)	3	0.004	^***^	0.003	^***^	<0.001	^***^	0.017	^***^	0.010	^***^
CG × SL	45	<0.001	^***^	<0.001	^***^	<0.001	^***^	0.002	^***^	0.005	^***^
Residuals	128	<0.001	–	<0.001	–	<0.001	–	<0.001	–	<0.001	–
Polyunsaturated fatty acid content	C18:2			C18:3		C20:2		C20:3		PUFA	
	DF	MS	*p*	MS	*p*	MS	*p*	MS	*p*	MS	*p*
Camelina genotype (CG)	15	0.004	^***^	0.010	^***^	<0.001	^***^	<0.001	^***^	0.009	^***^
Shade level (SL)	3	0.005	^***^	0.017	^***^	<0.001	^***^	<0.001	^***^	0.045	^***^
CG × SL	45	<0.001	^***^	0.003	^***^	<0.001	^***^	<0.001	^***^	0.004	^***^
Residuals	128	<0.001	–	<0.001	–	<0.001	–	<0.001	–	<0.001	–

C16:0 (palmitic acid); C18:0 (stearic acid); C18:1 (oleic acid); C18:2 (linoleic acid); C18:3 (linolenic acid); C20:1 (eicosenoic acid); C20:2 (eicosadienoic acid); C20:3 (eicosatrienoic acid); C22:1 (erucic acid); SFA (saturated fatty acid); MUFA (monounsaturated fatty acid); PUFA (polyunsaturated fatty acid); UFA/SFA ratio = The sum of unsaturated fatty acid/the sum of saturated fatty acid; MUFA/PUFA ratio = The sum of mono-unsaturated/the sum of poly-unsaturated.

*** represents significant at 0.001 probability level.

### Determination of shade tolerance of camelina genotypes using membership function value

To evaluate the level of shade tolerance of the 16 camelina genotypes tested, shade tolerance index (STI) of each camelina genotype under LST, MST, and HST was determined by considering both comprehensive shade tolerance coefficient (CSTC) and seed yield shade tolerance coefficient (YSTC) [Eq. (3–5)] for each experimental year (Table 4). While the different patterns of STI values were shown for the camelina genotypes tested for the other shade treatments, overall, the mean STI values of camelina genotypes across the three treatments, including CamK7 (1.29 and 1.10 for 2020–2021 and 2021–2022, respectively), CamK9 (2.06 and 1.66 for 2020–2021 and 2021–2022, respectively), CamK10 (1.08 and 1.07 for 2020–2021 and 2021–2022, respectively), CamC2 (1.24 and 0.84 for 2020–2021 and 2021–2022, respectively), CamC4 (1.00 and 1.49 for 2020–2021 and 2021–2022, respectively), and ‘SO-40’ (1.26 and 1.57 for 2020–2021 and 2021–2022, respectively) were consistently greater than other camelina genotypes for each year (Table 4). Among them, CamK7, CamK9, Cam C4, and ‘SO-40’ showed relatively greater STI values. In contrast, CamK1 and CamC1 showed lower STI values for each treatment across the two experimental years compared to other camelina genotypes.

**Table 4:** Comprehensive shade tolerance coefficient (CSTC), seed yield shade tolerance coefficient (YSTC), and shade tolerance index (STI) of camelina genotypes under low shade treatment (LST), medium shade treatment (MST), and high shade treatment (HST) in 2020–2021 and 2021–2022.

Year	Genotypes	LST			MST			HST			Mean
		CSTC	YSTC	STI	CSTC	YSTC	STI	CSTC	YSTC	STI	STI
2020–2021	CamK1	0.94	0.62	0.59	0.93	0.77	0.71	0.62	0.22	0.13	0.48
	CamK2	0.90	0.69	0.62	0.89	1.26	1.12	0.79	0.57	0.44	0.73
	CamK3	1.01	1.10	1.11	0.99	0.74	0.73	0.90	0.75	0.68	0.84
	CamK4	1.07	0.93	1.00	1.08	0.65	0.71	1.07	0.98	1.05	0.92
	CamK5	1.16	1.08	1.24	0.95	0.68	0.64	0.98	1.04	1.01	0.97
	CamK6	0.90	0.31	0.28	1.01	1.02	1.03	0.93	0.41	0.38	0.56
	CamK7	1.16	0.88	1.03	0.91	0.97	0.88	0.96	1.36	1.31	1.29
	CamK8	0.94	0.74	0.70	0.87	1.31	1.14	0.94	1.04	0.98	0.94
	CamK9	1.09	1.42	1.54	1.02	2.13	2.17	1.11	2.22	2.47	2.06
	CamK10	1.14	1.19	1.35	1.04	1.41	1.46	0.92	0.46	0.43	1.08
	CamK11	1.05	0.86	0.90	0.89	0.45	0.41	1.07	1.65	1.77	1.02
	CamC1	1.02	0.72	0.73	1.07	0.73	0.78	0.86	0.68	0.58	0.70
	CamC2	1.08	1.56	1.68	0.88	0.87	0.76	0.78	1.64	1.28	1.24
	CamC3	1.15	1.07	1.22	0.98	0.32	0.32	0.85	1.08	0.92	0.82
	CamC4	1.04	1.05	1.09	1.03	1.03	1.06	0.92	0.94	0.87	1.00
	‘SO-40’	0.93	1.80	1.68	1.04	1.66	1.73	1.05	0.97	1.02	1.26
2021–2022	CamK1	0.94	0.91	0.86	0.87	0.69	0.60	0.94	0.77	0.72	0.73
	CamK2	1.13	0.93	1.05	0.94	0.67	0.63	1.01	0.61	0.62	0.77
	CamK3	1.04	0.84	0.87	0.85	0.72	0.61	0.91	1.01	0.91	0.80
	CamK4	0.92	0.65	0.60	1.00	0.72	0.72	0.91	0.66	0.60	0.64
	CamK5	0.94	0.91	0.85	0.98	1.01	0.99	0.95	1.41	1.34	1.06
	CamK6	1.08	1.28	1.39	0.93	1.04	0.96	1.04	1.04	1.09	1.15
	CamK7	1.02	1.22	1.24	0.87	0.96	0.83	1.11	1.10	1.22	1.10
	CamK8	0.93	0.63	0.58	0.89	1.04	0.93	0.86	0.69	0.59	0.70
	CamK9	1.01	2.15	2.17	0.98	1.31	1.29	0.93	1.62	1.51	1.66
	CamK10	0.94	1.17	1.10	0.89	1.37	1.22	0.85	1.05	0.89	1.07
	CamK11	1.09	1.15	1.25	1.15	0.83	0.95	1.01	0.94	0.96	1.05
	CamC1	0.83	0.12	0.10	0.92	0.62	0.57	0.96	0.45	0.43	0.37
	CamC2	1.00	0.89	0.89	0.90	0.78	0.70	0.90	1.02	0.92	0.84
	CamC3	0.96	0.84	0.81	0.97	0.85	0.83	1.00	0.71	0.71	0.78
	CamC4	1.14	1.25	1.43	1.19	1.61	1.91	1.08	1.05	1.14	1.49
	‘SO-40’	0.95	1.07	1.01	1.10	1.77	1.93	0.95	1.86	1.77	1.57

**Figure 5:**
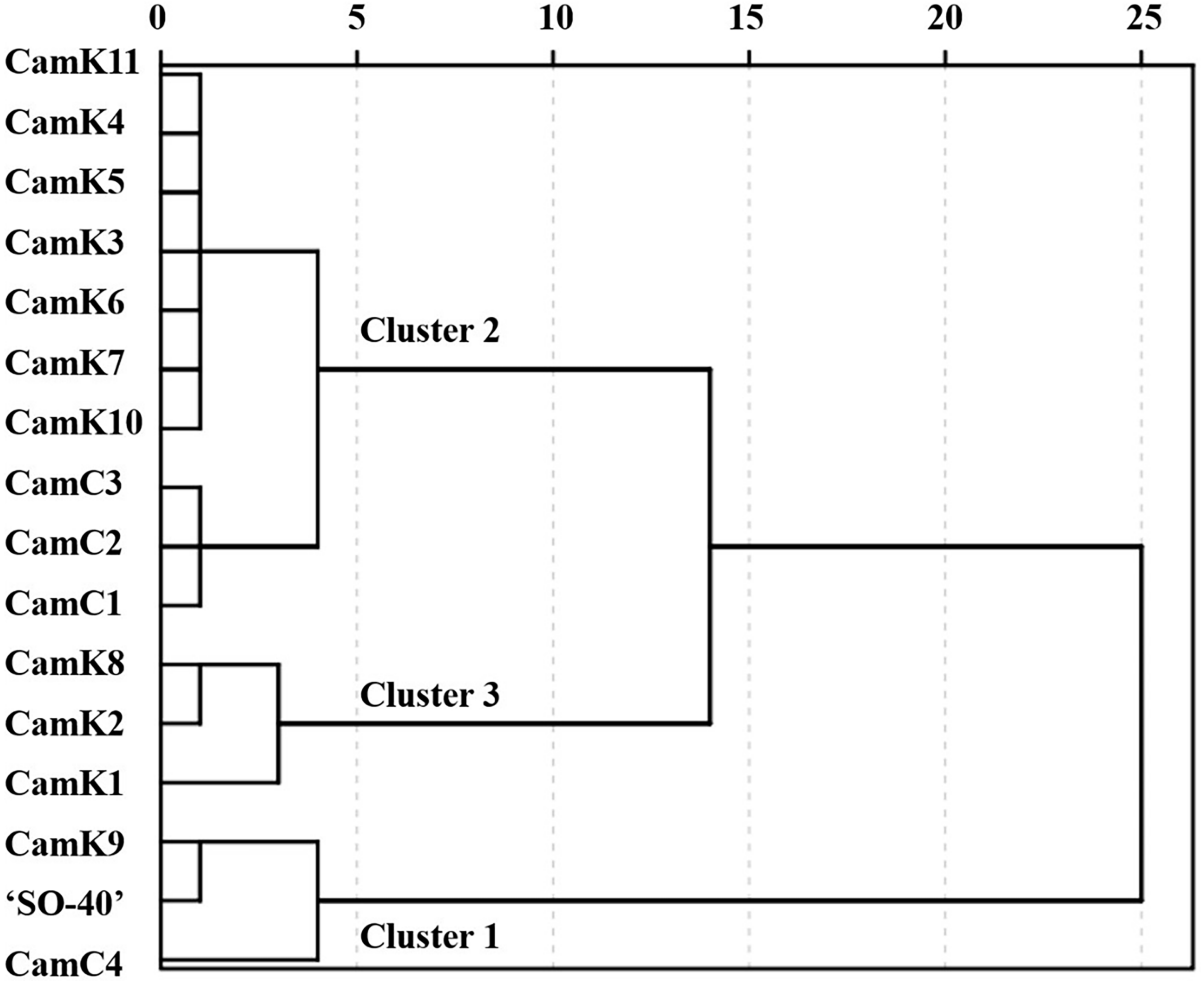
The dendrogram was constructed using the comprehensive tolerance membership values for the evaluation of shade tolerance of the 16 camelina genotypes. The camelina genotypes in Cluster 1, Cluster 2, and Cluster 3 represent the relatively shade-tolerant, medium shade-tolerant, and shade-sensitive camelina genotypes. The value of the comprehensive tolerance membership function for each camelina genotype was calculated considering the performance of each genotype under those three different shade levels (LST, MST, and HST) using the Eq. (8).

Firstly, to rank the shade tolerance levels of the 16 camelina genotypes, the comprehensive membership function [Eq. (7)] was applied considering the membership function value and weight of the measured parameters. The weights described in Supplementary Table 2 were calculated based on the correlation coefficient (r) between the shade tolerance coefficient (STC) [Eq. (2)] and the STI of each camelina genotype for each parameter measured. Then, the comprehensive performance of each camelina genotype under the different treatments was determined using Eq. (8). The dendrograms constructed using those values partitioned the 16 camelina genotypes into three groups (Figure 5). Across the treatments and experimental years, CamK9, CamC4, and ‘SO-40’ were classified into Cluster 1 with the greater comprehensive tolerance membership values of 1.35, 1.31, and 1.19, respectively, but distinctly separated from the camelina genotypes in Cluster 2 (CamK3-K7, CamK10-K11, and CamC1-C3 with the values ranging from 0.88 to 1.08) and Cluster 3 (Cam K1-K2 and CamK8 with the values ranging from 0.66 to 0.80). The greater comprehensive tolerance membership values for CamK9, CamC4, and ‘SO-40’ indicated that they are more shade-tolerant, whereas the camelina genotypes with the lower values were shade-sensitive.

## Discussion

Intercropping of camelina with pea (*Pisum sativum* L.) and soybean [*Glycine max* (L.) Merr.]-maize (*Zea mays* L.) systems has improved the farmland nutrient use efficiency and filled the gaps in grain legume, especially the legume with the poor establishment (Ross et al., 2004; Paulsen, 2007; Berti et al., 2017). Under low light or shaded conditions, understanding the changes of camelina photosynthetic, physiological, and lipid biosynthetic (i.e., saturated or unsaturated fatty acids) characteristics would help select or breed shade-tolerant camelina genotypes as the optional crop in an intercropping system. To the best of our knowledge, this study was the first to evaluate the shade tolerance of the 16 camelina genotypes under different artificial shade treatments. The results obtained from the study would provide the baseline information on the responses of camelina genotypes to the different shade treatments and help determine the suitable intercropped camelina genotypes.

As previously reported in rice (*Oryza sativa* L.; Chen et al., 2019) and an herb plant, *Pinellia ternata* (Xue et al., 2019), shade treatment significantly increased the photosynthetic pigment of the camelina plant, including total chlorophyll, chlorophyll *a*, and chlorophyll *b* contents (Figure 1). This is because under shade conditions, to capture more light energy, the camelina plants increase the chlorophyll density per unit of leaf area, thus causing the increase in photosynthetic pigment (Wittmann et al., 2001). While shade increased the content of the major photosynthetic pigments, it reduced the chlorophyll *a*/*b* ratio in camelina in this study. This result is in agreement with the previous study reported on rice (Chen et al., 2019) and tea plants (*Camellia sinensis* L.; Sano et al., 2020). Under shade conditions, the rate of synthesis and decomposition of chlorophyll *a* and chlorophyll *b* affect the ratio of chlorophyll *a*/*b* (Tanaka and Tanaka, 2011). It has been shown that by increasing the light-harvesting chlorophyll *b* proteins levels, the plants could promote photosynthesis and improve their adaptation (Sano et al., 2020). Thus, one explanation might be that the conversion of chlorophyll *a* to chlorophyll *b* in camelina leaves was accelerated under shade conditions, resulting in a greater rate of increase in chlorophyll *b* than chlorophyll *a*, eventually decreasing the ratio of chlorophyll *a*/*b*. The chlorophyll fluorescence parameter F_v_/F_m_ reflects the potential maximum quantum efficiency of photosystem II (PSII), which has been able to detect earlier effects of various stresses (i.e., herbicide, drought, waterlogging) on plants (Murchie and Lawson, 2013; Zhang et al., 2016; Bano et al., 2021). In this study, the Tukey *post-hoc* comparisons test showed that the shade at the current level reduced the F_v_/F_m_ values (Figure 1), indicating the sensitivity of camelina to the shade. Considering the similar reduction pattern between F_v_/F_m_ and seed yield (Figures 1, 3), it suggests that the F_v_/F_m_ could be applied as a valuable indicator to predict the potential camelina seed yield under shade conditions.

Shade avoidance is an effective strategy that plants have evolved in response to shade, which would help the plants to harvest more light and energy for photosynthesis. In this study, the camelina genotypes under shade treatment showed the typical shade avoidance traits with increased plant height (Figure 2). The similar results were also reported for shade-treated rice (Wu et al., 2017), soybean (Liu et al., 2015), and *Arabidopsis thaliana* (L.; Carabelli et al., 2018). The shade avoidance response triggered in our study might be due to the lower Red/Far-red ratio that resulted from the black netting used for shade treatment (Wollenberg et al., 2008; Chen et al., 2019).

In this study, LST had no significant effect on the camelina seed yield plant^−1^, indicating the camelina could adapt well to the low light conditions (about a 15% reduction in natural light intensity). In contrast, the camelina seed yield plant^−1^ was significantly reduced by MST and HST (>25% reduction in natural light intensity; Figure 3). This demonstrated that with increasing the shade level the adverse effect of shade on the seed yield of camelina increased accordingly. The decrease in camelina seed yield plant^−1^ was associated with reducing the number of silicles and branches plant^−1^ (Figure 2). While the reduced leaf area proved to be a disadvantage for the leaf to absorb more light (Figure 2), the increased photosynthetic pigments of the camelina crop under low shade treatment could compensate for the photosynthetic capacity with the high concentration of chloroplasts, eventually producing a similar seed yield as NST. However, under the relatively greater shade levels (MST and HST), the camelina seed yield plant^−1^ was significantly decreased even if the photosynthetic pigments were increased. A plausible explanation is that the effect of the greater shade level on the growth and development of camelina (i.e., reduced silicles and branch numbers) is beyond the compensation for seed yield through increasing the photosynthetic capacity. A previous study also reported although the chlorophyll content increased due to increased shade level, decreased active photosynthetic radiation caused a significant reduction in pod numbers and thus seed yield plant^−1^ in mungbean (*Vigna radiata* L.; Islam et al., 1993).

Accelerated flowering is also a manifestation of shade avoidance (Nozue et al., 2015), which has been observed on shade-treated *A. thaliana* (Wollenberg et al., 2008). Further molecular work revealed that under shade conditions, far-red enrichment could bypass *FLOWERING LOCUS C* (*FLC*)-mediated late flowering and induce early flowering (Wollenberg et al., 2008). Contrary to shade-treated *A. thaliana*, the present study showed that the mean flowering time of shade-treated camelina genotypes was delayed (Supplementary Figure 1). While the molecular mechanism on this is unknown and needs to be further investigated, delayed flowering in a forage crop, alfalfa (*Medicago sativa* L.) under shade conditions has been suggested by the downregulation of *SQUAMOSA PROMOTER BINDINGP ROTEIN LIKE 3* (*SPL3*) to promote flowering (Lorenzo et al., 2019). Additionally, the mean flowering duration across the 16 camelina genotypes under the current range of shade levels was shorter up to a week compared to the NST. Therefore, the shortened lifecycle and the aforementioned altered flowering phenology (i.e., first and end days of anthesis) resulting from the shade treatment could be attributed to the reduced camelina seed yield.

Light has been suggested to facilitate fatty acid synthesis in photosynthetic oilseeds by providing both ATP and carbon skeletons (Constantopoulos and Boloch, 1967; Willms et al., 1999). In this study, although the camelina genotypes for LST and NST showed similar values in the mean content of seed oil and different fatty acid groups, those values were significantly reduced for MST and HST (Figures 3, 4). This could probably be caused by the different light intensities that resulted from the other shade conditions. Under the relatively greater shade conditions (MST and HST), the reduced light intensity could probably reduce the photosynthetic ability far more than that of NST, which would further affect the supply and use of ATP and CO_2_ (Willms et al., 1999), eventually leading to the decrease in seed oil and unsaturated fatty acid group contents (monounsaturated and polyunsaturated fatty acids). Additionally, a reduction in the accumulation of unsaturated fatty acid due to the lower light intensity was reported in cyanobacteria (Ronda and Lele, 2008).

Interestingly, we found that HST significantly increased the total saturated fatty acid than the lower shade treatment (Figure 4). By affecting the expression levels of the three fatty acid desaturase genes, light promotes/restrains the conversion of fatty acids to unsaturated fatty acids (Kis et al., 1998; Sun et al., 2018). In the current study, while the exact molecular mechanisms for the decrease of unsaturated fatty acids or increase of saturated fatty acids under shade treatment are unknown, it is certain that the low light intensity resulting from the shade treatment had a negative effect on the fatty acid desaturase gene expression in camelina and thus affected the conversion of fatty acids to saturated or unsaturated fatty acids. Furthermore, the decrease in the ratio between monounsaturated and polyunsaturated fatty acids indicated that the camelina genotypes under relatively greater shade conditions could probably produce the seed oil with higher oxidative stability than that under lower shade conditions (Budin et al., 1995). Climatic differences (i.e., temperature) affected the ratio and concentrations between saturated and unsaturated fatty acids (Raziei et al., 2018). The cold climates have favored the accumulation of unsaturated fatty acids relative to the saturated fatty acids in camelina seed oil. In this study, the present finding may provide a novel agricultural production strategy by intercropping camelina into the crops with the taller canopy or greater shade level in an intercropping system to obtain the higher ratio of monounsaturated and polyunsaturated fatty acids for other industrial purposes (i.e., biofuel).

One of the primary purposes of this study was to determine the shade tolerance of the 16 camelina genotypes using an appropriate regression model with all parameters measured above and utilize the shade-tolerant genotypes in an intercropping agricultural system. Generally, although the shade tolerance for each camelina genotype varied among all genotypes tested, the evaluation showed that camelina is a relatively low shade tolerance endured crop species compared to other intercropped crops, such as alfalfa that could endure the shade level up to 50% natural light reduction with no obvious plant biomass loss (Gao et al., 2022b). Further, the dendrograms constructed using the comprehensive tolerance membership values calculated in this study showed that the three camelina genotypes, including CamK9, CamC4, and ‘SO-40’ were the relatively shade-tolerant genotypes among the camelina genotypes tested. These three camelina genotypes could grow under the shade level of up to 25% natural light reduction, producing the similar seed yield and oil quality. In the maize-soybean intercropping system, the mean natural light interception has been previously reported as about 40 ~ 50% (Wen et al., 2020). A previous study conducted in the U.S. Midwest (North Dakota) showed that intercropping a winter camelina ‘Jeolla’ into standing maize at V4-V5 or soybean at V3-V4 could avoid competition and maximize the crop yields of the intercropping system (Berti et al., 2017). Although the difference in shade tolerance might be presented between the camelina genotypes used in this study (spring type) and the winter camelina (Berti et al., 2017), the determined suitable dates for intercropping the winter camelina into the intercropping system still provides the reference valuable for the spring camelina genotypes. Our recent study demonstrated that fall-seeded the same genotypes, CamK9 and CamC4, at the same region as the current study were characterized as the potential high-yielding camelina genotypes (Gao et al., 2022b). Therefore, future studies are needed to optimize the intersowing management (i.e., suitable intersowing date, strategy for minimizing competition) to increase yields of both intercropped crops while enhancing the ecosystem services.

## Conclusion

This study was the first to evaluate the shade tolerance of the 16 spring camelina genotypes under different artificial shade levels (LST, MST, and HST), and subsequently used a modified comprehensive membership function to determine the shade-tolerant camelina candidates for the potential intercropped crop. Shade treatment significantly affected the photosynthetic and physiological parameters, seed production, and seed oil quality of the spring camelina genotypes. Overall, spring camelina is a relatively low shade-tolerant crop species, which could endure the shade level of up to 25% natural light reduction. Among the tested camelina genotypes, CamK9, CamC4, and ‘SO-40’ were the relative shade tolerance endured genotypes with the great potential to intercrop with maize or other small crops. However, there are still many unanswered questions on camelina intersowing management, including optimization of intersowing date, minimization of competition, and maximization of both crop yields needed to address. The present study provided the baseline information on the responses of camelina genotypes to the different shade treatments, which would help select shade-tolerant genotypes and thus contribute to the camelina breeding program.

## Data availability statement

The raw data supporting the conclusions of this article will be made available by the authors, without undue reservation.

## Author contributions

YW and C-JZ: conceptualization, methodology, data curation, writing—original draft preparation, software, and validation. YW, YG, ZL, and MC: visualization and investigation. YW, JY, YG, ZL, D-SK, MC, YF, HZ, XY, and C-JZ: writing—reviewing and editing. All authors contributed to the article and approved the submitted version.

## Funding

This work was mainly supported by National Science Foundation of China (Grant No. 32171670), Natural Science Fund for colleges and university of Jiangsu Province (Grant No. 19KJB180034), and Natural Science Foundation of Jiangsu Province (Grant No. BK20190895). Funding was also provided by Major Focus Projects of Henan Academy of Sciences (Grant No. 190113004), High-level Innovation and Entrepreneurship Talents Introduction of Jiangsu, and High-level Talents program of Lv-Yang-Jin-Feng of Yangzhou for analyzing camelina seed oil content and fatty acid composition.

## Conflict of interest

YF is employed by Henan Napu Biotechnology Co., Ltd.

The remaining authors declare that the research was conducted in the absence of any commercial or financial relationships that could be construed as a potential conflict of interest.

## Publisher’s note

All claims expressed in this article are solely those of the authors and do not necessarily represent those of their affiliated organizations, or those of the publisher, the editors and the reviewers. Any product that may be evaluated in this article, or claim that may be made by its manufacturer, is not guaranteed or endorsed by the publisher.

## Footnotes

^1^ http://data.cma.cn/

## Supplementary material

SUPPLEMENTARY FIGURE 1 Phenogram illustration of the mean first day of anthesis and flowering duration of camelina across the 16 camelina genotypes to different shade treatments in 2020–2021 **(A)** and 2021–2022 **(B)**.
