# Supplementary figures and images for: Agronomic evaluation of shade tolerance of 16 spring Camelina sativa (L.) Crantz genotypes under different artificial shade levels using a modified membership function
Source: Front Plant Sci. 2022 Aug 29;13:978932. doi: 10.3389/fpls.2022.978932 (PMC9465330; doi:10.3389/fpls.2022.978932)

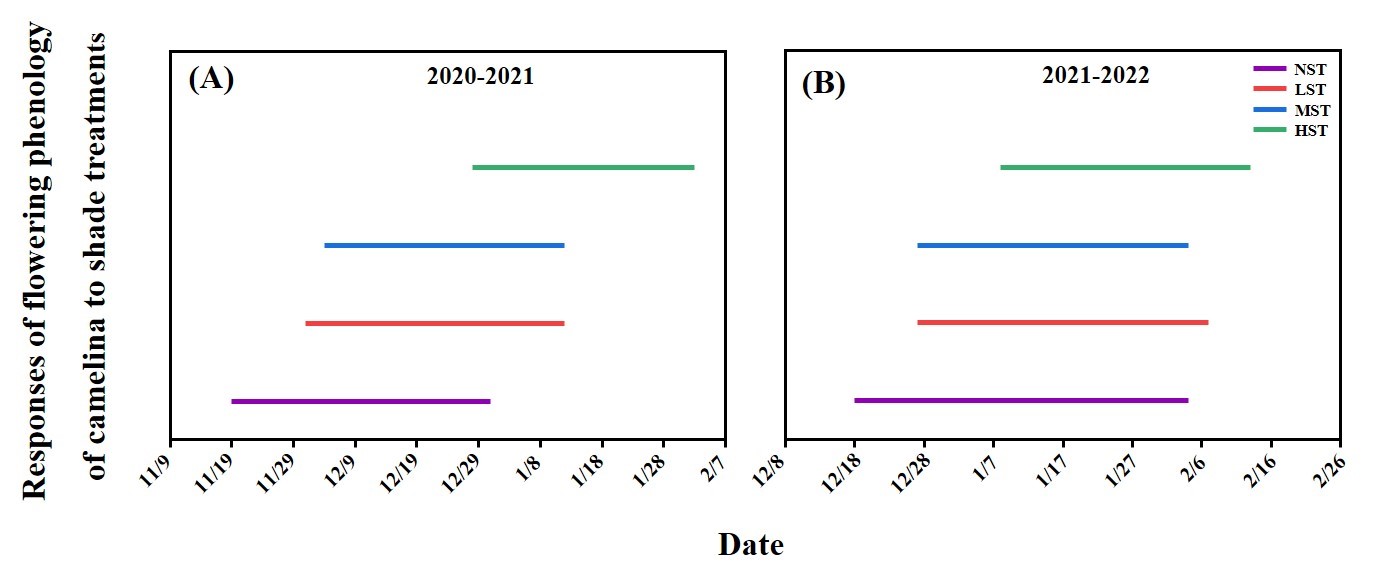

Supplement: SUPPLEMENTARY FIGURE 1 — Phenogram illustration of the mean first day of anthesis and flowering duration of camelina across the 16 camelina genotypes to different shade treatments in 2020–2021 (A) and 2021–2022 (B). [file Image_1.JPEG]
