# Supplementary material for: Agronomic evaluation of shade tolerance of 16 spring Camelina sativa (L.) Crantz genotypes under different artificial shade levels using a modified membership function
Source: Front Plant Sci. 2022 Aug 29;13:978932. doi: 10.3389/fpls.2022.978932 (PMC9465330; doi:10.3389/fpls.2022.978932)
Supplement: Supplementary file 2 [file Data_Sheet_1.docx]

**SUPPLEMENTARY TABLE 1** The total yearly light intensity and mean temperature under different shade treatments during the study in 2020-2022 and precipitation data in experimental site.

| **2020-2022** | **Total yearly light intensity ^a^ (lum/ft²)** | | | |  | **Mean temperature (°C)** | | | | **Precipitation (mm)** | | |
| --- | --- | --- | --- | --- | --- | --- | --- | --- | --- | --- | --- | --- |
|  | **NST** | **LST** | **MST** | **HST** |  | **NST** | **LST** | **MST** | **HST** | **2020-2021** | **1981-2010 ^b^** |  |
| Oct.9-24 | 3394.4 | 2931.3 | 2685.7 | 1708.4 |  | 19.4 | 19.5 | 19.5 | 18.1 | 21.6 | 28.6 |  |
| Oct.25-Nov.9 | 3632.0 | 3144.0 | 2845.8 | 1819.3 |  | 16.1 | 16.0 | 16.0 | 15.7 | 16.6 | 19.7 |  |
| Nov.10-24 | 3607.6 | 3032.9 | 2384.4 | 1633.6 |  | 14.0 | 13.7 | 14.1 | 13.7 | 22.6 | 24.6 |  |
| Nov.25-Dec.9 | 2839.6 | 2284.2 | 2053.4 | 1369.9 |  | 8.8 | 7.9 | 8.4 | 6.8 | 27.3 | 33.4 |  |
| Dec.10-24 | 2508.0 | 2061.0 | 1871.4 | 1022.0 |  | 5.4 | 6.3 | 5.2 | 5.0 | 24.8 | 19.8 |  |
| Dec.24-Jan.8 | 2918.6 | 2493.3 | 2195.0 | 1006.0 |  | 11.0 | 10.1 | 8.4 | 7.9 | 22.5 | 20.2 |  |
| Mean/Sum | 18900.2 | 15946.8 | 14035.7 | 8559.1 |  | 12.5 | 12.3 | 11.9 | 11.2 | 135.4 | 146.3 |  |

^a^ NST, LST, MST and HST represent no shade treatment, low shade treatment, medium shade treatment and high shade treatment, respectively.

^b^ The past 30-year mean precipitation data were obtained from Yangzhou Meteorological Administration.

**SUPPLEMENTARY TABLE 2** The correlation coefficients (r) and weight of parameters measured under low shade treatment (LST), medium shade treatment (MST) and high shade treatment (HST) in 2020-2021 and 2021-2022.

| Year | Parameter | LST |  |  | MST |  |  | HST |  |
| --- | --- | --- | --- | --- | --- | --- | --- | --- | --- |
|  |  | r ^a^ | Weight |  | r | Weight |  | r | Weight |
| 2020-2021 | Chl T | 0.413 | 0.06 |  | 0.031 | 0.01 |  | 0.359 | 0.04 |
|  | Chl a | 0.366 | 0.05 |  | 0.131 | 0.02 |  | 0.377 | 0.04 |
|  | Chl b | 0.434 | 0.06 |  | -0.303 | 0.06 |  | 0.078 | 0.01 |
|  | Chl a/b | -0.190 | 0.03 |  | 0.363 | 0.07 |  | 0.032 | 0.00 |
|  | Fv/Fm | 0.056 | 0.01 |  | 0.257 | 0.05 |  | 0.316 | 0.03 |
|  | PH | 0.576* | 0.09 |  | 0.110 | 0.02 |  | 0.505* | 0.05 |
|  | LA | 0.175 | 0.03 |  | 0.226 | 0.04 |  | 0.433 | 0.05 |
|  | BN | 0.614* | 0.09 |  | 0.556* | 0.10 |  | 0.695* | 0.07 |
|  | SN | 0.479 | 0.07 |  | 0.443 | 0.08 |  | 0.508* | 0.05 |
|  | SY | 0.353 | 0.05 |  | 0.462 | 0.08 |  | 0.551* | 0.06 |
|  | SOC | -0.519* | 0.08 |  | 0.016 | 0.00 |  | -0.307 | 0.03 |
|  | C16:0 | 0.216 | 0.03 |  | 0.021 | 0.00 |  | 0.759** | 0.08 |
|  | C18:0 | 0.116 | 0.02 |  | -0.162 | 0.03 |  | 0.288 | 0.03 |
|  | C18:1 | -0.028 | 0.00 |  | 0.423 | 0.08 |  | 0.376 | 0.04 |
|  | C18:2 | -0.192 | 0.03 |  | 0.200 | 0.04 |  | 0.012 | 0.00 |
|  | C18:3 | 0.109 | 0.02 |  | -0.234 | 0.04 |  | 0.312 | 0.03 |
|  | C20:1 | 0.260 | 0.04 |  | -0.026 | 0.00 |  | 0.563* | 0.06 |
|  | C20:2 | 0.335 | 0.05 |  | -0.201 | 0.04 |  | 0.198 | 0.02 |
|  | C20:3 | 0.416 | 0.06 |  | 0.081 | 0.01 |  | 0.379 | 0.04 |
|  | C22:1 | 0.165 | 0.02 |  | -0.160 | 0.03 |  | 0.306 | 0.03 |
|  | SFA | 0.191 | 0.03 |  | -0.166 | 0.03 |  | -0.050 | 0.01 |
|  | MUFA | 0.159 | 0.02 |  | 0.198 | 0.04 |  | 0.558* | 0.06 |
|  | PUFA | 0.011 | 0.00 |  | -0.194 | 0.04 |  | 0.333 | 0.04 |
|  | U/S ratio | 0.220 | 0.03 |  | -0.060 | 0.01 |  | 0.598* | 0.06 |
|  | MU/PU ratio | 0.110 | 0.02 |  | 0.427 | 0.08 |  | 0.543* | 0.06 |
| 2021-2022 | Chl T | 0.523* | 0.16 |  | 0.569* | 0.17 |  | 0.079 | 0.04 |
|  | Chl a | 0.545* | 0.17 |  | 0.248 | 0.07 |  | 0.033 | 0.01 |
|  | Chl b | 0.151 | 0.05 |  | 0.445 | 0.13 |  | 0.134 | 0.06 |
|  | Chl a/b | 0.222 | 0.07 |  | -0.253 | 0.08 |  | -0.016 | 0.01 |
|  | Fv/Fm | 0.119 | 0.04 |  | 0.315 | 0.09 |  | 0.180 | 0.08 |
|  | PH | 0.141 | 0.04 |  | 0.238 | 0.07 |  | 0.400 | 0.18 |
|  | LA | -0.090 | 0.03 |  | 0.157 | 0.05 |  | 0.505* | 0.22 |
|  | BN | 0.027 | 0.01 |  | 0.224 | 0.07 |  | -0.666** | 0.30 |
|  | SN | 0.528* | 0.17 |  | 0.269 | 0.08 |  | 0.154 | 0.07 |
|  | SY | 0.311 | 0.10 |  | 0.139 | 0.04 |  | 0.007 | 0.00 |
|  | SOC | 0.536* | 0.17 |  | 0.561* | 0.16 |  | 0.082 | 0.04 |

^a^ r is the correlation coefficient between the shade tolerance coefficient of the indicators (STC) and shade tolerance index (STI) of sixteen camelina genotypes.
